# Anxiolytic effects of *Enterococcus faecalis* 2001 on a mouse model of colitis

**DOI:** 10.1038/s41598-024-62309-3

**Published:** 2024-05-21

**Authors:** Kohei Takahashi, Minoru Tsuji, Osamu Nakagawasai, Kazuya Miyagawa, Kazuhiro Kurokawa, Atsumi Mochida-Saito, Masahiro Iwasa, Hiroyuki Iwasa, Shigeo Suzuki, Hiroshi Takeda, Takeshi Tadano

**Affiliations:** 1 Department of Pharmacology, School of Pharmacy, International University of Health and Welfare, 2600-1 Kitakanemaru, Ohtawara, Tochigi 324-8501 Japan; 2 Division of Pharmacology, Faculty of Pharmaceutical Sciences, Tohoku Medical and Pharmaceutical University, 4-4-1 Komatsushima, Aoba-Ku, Sendai, Miyagi 981-8558 Japan; 3 Nihon Berm Co., Ltd., 16-12, Nihonbashi-Kodenmacho, Chuo-Ku, Tokyo, 103-0001 Japan; 4 Department of Pharmacology, School of Pharmacy at Fukuoka, International University of Health and Welfare, 137-1 Enokizu, Okawa, Fukuoka 831-8501 Japan; 5 Department of Environment and Preventive Medicine, Graduate School of Medicine Sciences, Kanazawa University, 13-1 Takaramachi, Kanazawa, Ishikawa 920-8640 Japan

**Keywords:** Anxiety, BDNF, Corticosterone, Colitis, Drebrin, EF-2001, Anxiety, Ulcerative colitis, Animal disease models, Prefrontal cortex, Neurotrophic factors, Bacteria

## Abstract

Ulcerative colitis (UC) is a refractory inflammatory bowel disease, which is known to cause psychiatric disorders such as anxiety and depression at a high rate in addition to peripheral inflammatory symptoms. However, the pathogenesis of these psychiatric disorders remains mostly unknown. While prior research revealed that the *Enterococcus faecalis* 2001 (EF-2001) suppressed UC-like symptoms and accompanying depressive-like behaviors, observed in a UC model using dextran sulfate sodium (DSS), whether it has an anxiolytic effect remains unclear. Therefore, we examined whether EF-2001 attenuates DSS-induced anxiety-like behaviors. Treatment with 2% DSS for seven days induced UC-like symptoms and anxiety-like behavior through the hole-board test, increased serum lipopolysaccharide (LPS) and corticosterone concentration, and *p*-glucocorticoid receptor (GR) in the prefrontal cortex (PFC), and decreased *N*-methyl-d-aspartate receptor subunit (NR) 2A and NR2B expression levels in the PFC. Interestingly, these changes were reversed by EF-2001 administration. Further, EF-2001 administration enhanced CAMKII/CREB/BDNF-Drebrin pathways in the PFC of DSS-treated mice, and labeling of p-GR, p-CAMKII, and p-CREB showed colocalization with neurons. EF-2001 attenuated anxiety-like behavior by reducing serum LPS and corticosterone levels linked to the improvement of UC symptoms and by facilitating the CAMKII/CREB/BDNF-Drebrin pathways in the PFC. Our findings suggest a close relationship between UC and anxiety.

## Introduction

Ulcerative colitis (UC) is a refractory inflammatory bowel disease (IBD) that affects approximately 1.4 million people in the United States and 2.2 million people in Europe. Patients with IBD, including UC and Crohn’s disease, have been reported to develop psychiatric disorders such as obsessive–compulsive disorder, panic disorder, anxiety, depression, and autism at a high rate^1–5^; however, the mechanism of pathogenesis of these disorders remains unclear.

The composition of the intestinal microbiota in patients with IBD differs from that of healthy individuals, resulting in an imbalance between gram-positive and gram-negative bacteria^6^. Lipopolysaccharide (LPS), a component of the outer membrane of gram-negative bacteria, may be transferred into the bloodstream in IBD due to increased permeability of the intestinal cell membrane following enteritis symptoms^6–8^. In animal studies, intraperitoneal administration of LPS has been reported to act on the adrenal cortex and increase blood corticosterone levels, resulting in anxiety-like behavior^9–11^. Hyperactivation in the brain of the glucocorticoid receptor (GR), a receptor for corticosterone, is implicated in the development of anxiety-like behavior^12–15^. From these findings, it can be surmised that gut microbiota imbalance may adversely affect the brain through elevated serum LPS and corticosterone, leading to anxiety symptoms.

Activation of GR in the ventral hippocampus and prefrontal cortex (PFC) suppresses both the expression of *N*-methyl-d-aspartate receptor (NMDAR) subunit (NR) 2A and NR2B as well as anxiety-like behavior^16–22^. NMDAR is composed of five subunits (NR1, NR2A, NR2B, NR2C, and NR2D) and the absence of these subunits results in reduced phosphorylation levels of the calcium/calmodulin-dependent protein kinase (CAMK) II/cAMP-responsive element-binding protein (CREB) pathway^23–25^. Phosphorylation of CREB regulates the expression of brain-derived neurotrophic factor (BDNF) and drebrin, which are involved in synaptic plasticity; studies have reported these proteins to be implicated in anxiolytic effects^23,26,27^. These findings suggest that GR inactivation may have an anxiolytic effect by activating synaptic plasticity.

Dextran sulfate sodium (DSS), which causes inflammatory changes in the intestinal mucosa similar to UC, has shown to induce depression-like and anxiety-like behaviors^28–32^. Moreover, DSS-treated mice exhibit the disruption of gut microbiota balance, decreased intestinal barrier integrity, and elevated serum LPS, as well as elevated serum corticosterone (cortisol in humans) observed in patients with anxiety^29,31,33–35^. Therefore, we considered that this model may be useful in the study of psychiatric disorders associated with IBD.

*Enterococcus faecalis 2001* (EF-2001) is a probiotic lactic acid bacterium used as a biological response modifier (BRM), produced by heat treating live *E. faecalis* 2001. EF-2001 is a unique strain of *E. faecalis* with no other strains identical to *E. faecalis* based on the Multilocus Sequence Typing analysis method; it has been reported to exhibit anti-inflammatory and anti-tumor effects through immune function modulation^32,36–40^. Previous studies demonstrated that EF-2001 reduced DSS-induced IBD-like symptoms and depression-like behaviors, as well as olfactory bulbectomy-induced depression-like behaviors and memory deficits^32,41,42^. However, the effect of EF-2001 on colitis-induced anxiety is unclear.

Against this background, and addressing the abovementioned gap in prior knowledge, we investigated whether EF-2001 attenuates DSS-induced changes in anxiety-like behavior and peripheral symptoms, and examined the molecular mechanisms underlying these effects.

## Materials and methods

All experiments were approved by the Ethics Committee of Animal Experiments at the International University of Health and Welfare (Ohtawara, Japan; approval number: 19015, 22009). All animal experiments complied with the ARRIVE guidelines and were performed in accordance with the guidelines established by the Animal Research Committee of the International University of Health and Welfare and the United States National Institutes of Health Guide for the Care and Use of Laboratory Animals. Efforts were made to minimize the suffering of the animals and reduce the number of animals used in the experiments. Measurements of all experiments and analyses were performed in a blinded manner.

### Animals

We used male ddY mice (age, 6–7 weeks; weight, 26–28 g; Japan SLC, Shizuoka, Japan) for all the experiments (total: n = 281; behavioral tests: n = 221; western blot analysis: n = 30; enzyme-linked immunosorbent assay (ELISA) analysis: n = 24; immunohistochemical study: n = 6). The mice were housed in cages containing five to six animals, and subjected to steady conditions (i.e., temperature, 23 ± 1 °C; humidity, 55 ± 5%, and 12/12 h light–dark cycle with lights on at 7:00). All behavioral tests were performed between 10:00 and 17:00. Each animal was tested only once for each behavioral test. The behavioral tests were conducted by an observer blinded to other study information. Mice were euthanized by cervical dislocation by skilled personnel, except for mice from which samples were collected for western blot, ELISA, and immunohistochemical studies.

### Drugs and treatments

Commercially available heat-treated EF-2001 was originally isolated from healthy human feces. It was supplied as a heat-killed, dried powder by Nihon Berm Co., Ltd. (Tokyo, Japan). DSS (1.5%, 2%, or 2.5%; Wako Pure Chemical Industries Ltd., Osaka, Japan) and EF-2001 (250 mg/kg) were dissolved in drinking water. Mice were given drinking water containing DSS ad libitum for seven days to induce colitis. Diazepam (DZP; 1 mg/kg; Wako Pure Chemical Industries Ltd.) and tandospirone (TDS; 1 or 3 mg/kg; Tokyo Chemical Industry Co., Ltd., Tokyo, Japan) were dissolved in 0.5% Tween-20 (Vehicle; Wako Pure Chemical Industries Ltd.). EF-2001 was administered orally (per os [p.o.]) from 14 days before the beginning of DSS administration until the day prior to the last DSS treatment. DZP and TDS were administered intraperitoneally (i.p.) 30 min before the behavioral tests on day seven after the start of DSS administration. The dose for each drug used was calculated from previous reports^32,41–44^.

### Evaluation of colon inflammation

**Figure 1:**
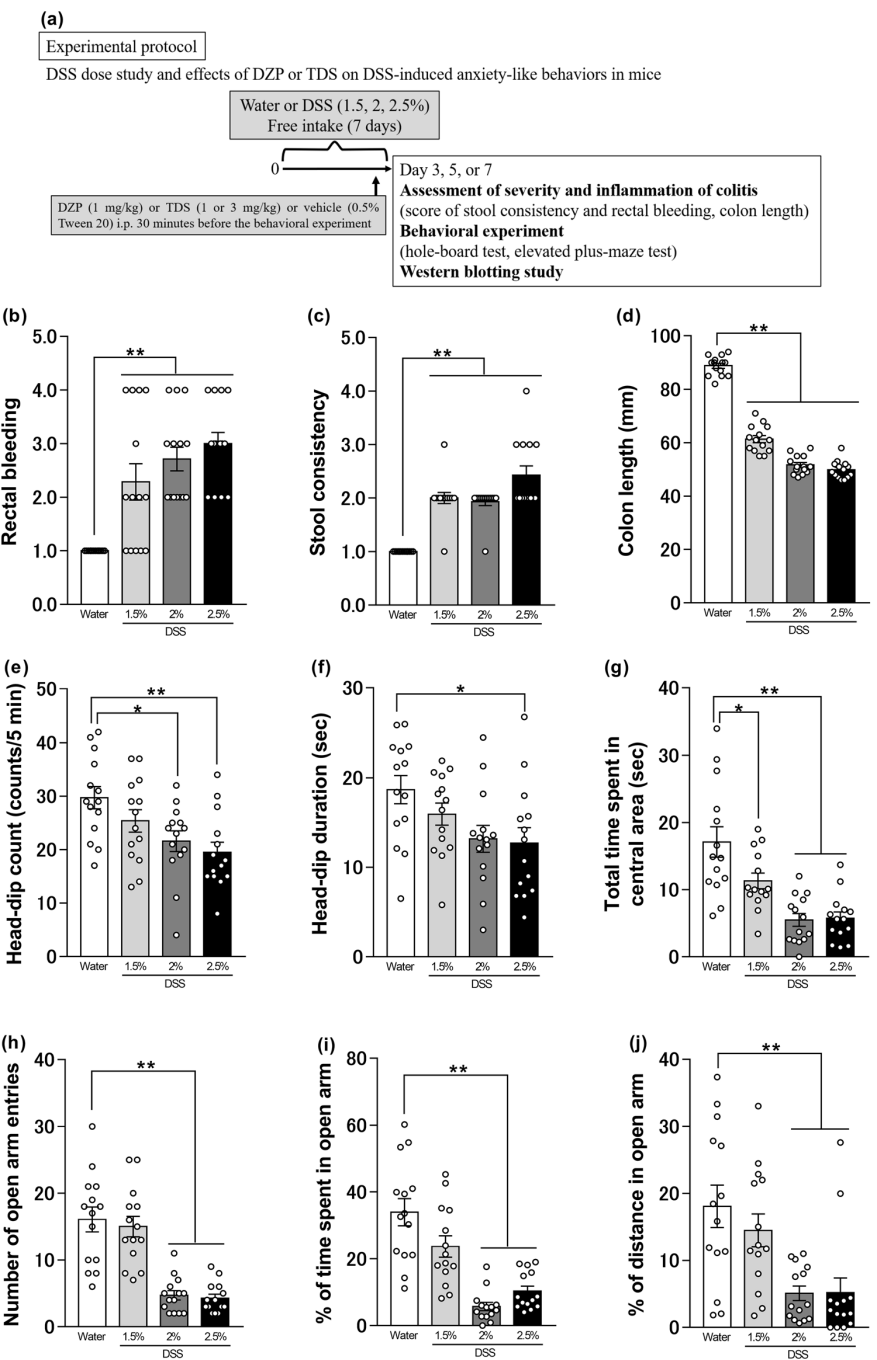
Effect of DSS concentration on ulcerative colitis-like findings and anxiety-like behaviors. (**a**) Experimental time course for assessment of inflammation, behavioral tests, western blotting, ELISA, and immunohistochemical of experimental protocols. Changes in rectal bleeding (**b**), stool consistency (**c**), colon length (**d**), head-dip counts (**e**), head-dip duration (**f**), total time spent in the central area (**g**), number of open-arm entries (**h**), percentage of distance traveled (**i**) or time spent (**j**) in the open arm in dextran sulfate sodium (DSS)-treated mice on day seven. Bars represent means ± standard error of mean (SEM). *p < 0.05 and **p < 0.01 vs. water group (n = 14 per group).

This evaluation was conducted according to the experimental protocol shown in Figs. 1a and 5a. Disease Activity Index (DAI) scores correlate well with pathological findings in the DSS-induced IBD model^45^. DAI scores were obtained based on methods described in prior publications^30–32,46^. DAI is a score for each of stool consistency and bleeding, as detailed in Supplemental Table S1. The length of the colon, from above the anus to the top of the cecum, was measured after the mice had been euthanized.

### Hole-board test

The hole-board test was carried out according to the method reported in prior research^47–49^. To investigate the changes in anxiety-like behaviors, the exploratory behaviors of mice on the hole-board, that is, the number or duration of head-dips, total time spent in the central area, and moving distance, were tested using an automatic hole-board apparatus (model ST-1; Muromachi Kikai, Tokyo, Japan). The apparatus consisted of a gray wooden box (50 cm × 50 cm × 50 cm) with four equidistant holes 3 cm in diameter in the floor^50^. Each animal was placed in the center of the hole-board and allowed to freely explore the apparatus over a 5-min period. Experiments were recorded with a video camera through a custom designed interface (DV-Trach; Muromachi Kikai). All of the data were analyzed and stored in a personal computer using analytical software (Comp ACT HBS; Muromachi Kikai).

### Elevated plus-maze test

To investigate changes in anxiety-like behavior, mice were tested using the elevated plus-maze paradigm (EPM-04M; Muromachi Kikai). The apparatus was elevated 40 cm from the ground and the maze consisted of two opposing open arms (30 cm × 6 cm × 0.3 cm) and two opposing enclosed arms (30 cm × 6 cm × 15 cm) that were connected by a central platform (6 cm × 6 cm, 70 lx), thus forming the shape of a plus sign. Each mouse was placed on a left front corner, and the distance that the mouse moved in the apparatus was recorded for 5 min by an overhead color CCD camera that tracked the center of the mouse. Moreover, the number of entries into and the time spent in open or enclosed arms were also recorded. Data from the CCD camera were collected through a custom designed interface (CAT-10; Muromachi Kikai) as a reflection signal. All of the data were analyzed and stored in a personal computer using analytical software (Comp ACT HBS; Muromachi Kikai). The results were calculated as mean ratios of the time spent or distance in the open arms to the total time spent or distance in both the open and enclosed arms. Moreover, the total number of entries into the open arms was also measured.

### Western blotting

Western blotting was performed according to the experimental protocol shown in Figs. 1a and 5a. Brain samples were collected from mice on day seven of DSS treatment. Moreover, mice were treated with water or EF-2001 for 20 days and sacrificed by decapitation 24 h after the last administration. The brain was immediately removed and the PFC, hypothalamus, amygdala, or ventral hippocampus were dissected quickly by a mouse brain slicer (Muromachi Kikai, Tokyo, Japan) to produce coronal sections of 1 mm thickness. We used samples from mice that had not undergone any behavioral tests. The brain atlas of Paxinos and Franklin was used as a reference to guide all dissections^51^. Protein isolation and western blotting were performed as described in prior works^52–54^. After electrophoresis, proteins were transferred electrically from the gel onto a polyvinylidene difluoride membrane using a semi-dry blotting apparatus (Bio-Rad Laboratories, Hercules, CA, USA). The blots were blocked for 30 min with 5% skim-milk in Tris-buffered saline supplemented with 0.01% Tween-20 (TBST). Subsequently, membranes were cut between 50 and 75 kDa, and probed with antibodies except for p-CAMKII, t-CAMKII, and BDNF against phosphorylated (p)-GR (Ser211) (1:1000; Cell Signaling Technology, Danvers, MA, USA, #4161), total (t)-GR (1:200; Santa Cruz Biotechnology, Dallas, TX, USA, sc-393232), NR1 (1:200; Cell Signaling Technology, #4204), NR2A (1:200; Cell Signaling Technology, #4205), NR2B (1:500; Cell Signaling Technology, #4207), p-CAMKII (1:1000; Cell Signaling Technology, #3361), t-CAMKII (1:1000; Cell Signaling Technology, #3362), p-CREB (1:500; Cell Signaling Technology, #9198), t-CREB (1:1000; Cell Signaling Technology, #9197), BDNF (1:1000; Abcam Ltd., Cambridge, UK, ab108319), synaptophysin (1:2000; Sigma-Aldrich, St. Louis, USA, S5768), synaptosomal-associated protein 25 (SNAP25; 1:1000; Santa Cruz Biotechnology, sc-7539), postsynaptic density protein 95 (PSD95; 1:1000; Cell Signaling Technology, #3450), drebrin (1:200; Medical & Biological Laboratories Co., Ltd., Tokyo, Japan, D029-3), microtubule-associated protein 2 (MAP2; 1:2000; Sigma-Aldrich, M9942), neuronal nuclei (NeuN; 1:500; Millipore, MAB377), and β-actin (1:1000; Santa Cruz Biotechnology, sc-47778) overnight at 4 °C. The blots were washed several times and then incubated at room temperature for 1 h with a secondary antibody (horseradish peroxidase-conjugated anti-rabbit, anti-goat, or anti-mouse immunoglobulin G (IgG) antibody diluted 1:10,000 with TBST containing 5% skim-milk). Blots were developed using an enhanced chemiluminescence assay kit (GE Healthcare, Buckinghamshire, UK) or ImmunoCruz (Santa Cruz Biotechnology) and scanned, optimized, and analyzed using the Quantity One 1-D Analysis Software version 4.5.2 (Bio-Rad Laboratories). The density of the corresponding bands was analyzed using Image Studio Lite version 5.2 (LI-COR Biosciences, Lincoln, NE, USA).

### Measurement of serum LPS and corticosterone concentration

ELISA was performed according to the experimental protocol shown in Fig. 5a. Mice were treated with water or EF-2001 for 20 days and decapitated 24 h after the last treatment. The blood samples were collected from the decapitated mice into spitz tubes for serum isolation (Eiken Chemical Co., Ltd., Tokyo, Japan, #HC1100), centrifuged at 4 °C, 15 min, 2380 × *g*, and the supernatant was collected as a serum sample and stored at − 80 °C until the day of measurement. LPS and corticosterone concentrations were quantified using the Mouse Anti-LPS IgG Antibody Assay Kit (Chondrex, USA, WA, #6106) and Corticosterone ELISA Kit AssayMax (AssayPro, USA, MO, #EC3001-1), respectively.

### Immunohistochemical study

Immunohistochemical study was performed according to the experimental protocol shown in Fig. 5a. Mice were sacrificed 24 h after the last administration of EF-2001. Brain samples were collected as described in prior works^41,42,55,56^. The brains were cut into 50-μm sections from the bregma − 1.40 mm to − 2.00 mm using a cryostat (Leica CM3050, Leica Biosystems, Tokyo, Japan).

Frozen sections were mounted on glass slides (Matsunami Glass, Osaka, Japan). After washing three times every 5 min, the sections were incubated with PBS containing 1% normal goat serum (Life Technologies Corporation, Carlsbad, CA, USA) or 3% bovine serum albumin and 0.3% Triton X-100 (PBSGT or PBSBT) at room temperature (23 ± 1 °C) for 2 h. The sections were incubated overnight at 4 °C with rabbit anti-p-GR (Ser211) (1:200; Cell Signaling Technology, #4161), rabbit anti-p-CAMKII (1:500; Cell Signaling Technology, #3361), rabbit anti-p-CREB (1:200; Cell Signaling Technology, #9198), mouse anti-NeuN (1:200; Millipore, MAB377), mouse anti-glial fibrillary acidic protein (GFAP; 1:200; Millipore, MAB360), or goat anti-ionized calcium-binding adaptor molecule 1 (Iba1; 1:200, Abcam, ab5076). Sections were washed and incubated overnight at 4 °C with goat anti-rabbit IgG Alexa Fluor 488 (1:200; Molecular Probes, Eugene, OR, USA, A-11008), goat anti-mouse IgG Alexa Fluor 568 (1:200; Molecular Probes, A-11004), Cy3-conjugated donkey anti-goat IgG (1:200; Jackson ImmunoResearch Inc., PA, West Grove, USA, 705-165-147), or FITC-conjugated donkey anti-rabbit IgG (1:200; Jackson ImmunoResearch, 711-095-152) with PBSGT or PBSBT, and with 4′,6-diamidino-2-phenylindole (DAPI, 1:100; Wako Pure Chemical Industries, Ltd) to identify the nuclei. Finally, sections were washed and covered with a cover slip with VECTASHIELD Mounting Medium (Vector Laboratories, Newark, CA, USA). Labeled sections were analyzed using a confocal laser-scanning microscope (FV1200; OLYMPUS, Tokyo, Japan).

### Statistical analysis

The results of the experiments are expressed as mean ± standard error of the mean (SEM). The statistical significance of differences was determined using Student's t-test for two-group comparisons. The significance of differences was determined via one- or two-way analysis of variance (ANOVA), followed by the Tukey–Kramer test or Bonferroni test for multiple-group comparisons. The statistical significance of differences in DAI scores was assessed using a non-parametric Mann–Whitney test for two-group comparisons or a non-parametric Kruskal–Wallis test followed by the Dunn test for multiple-group comparisons. The criterion of significance was set at p < 0.05. When the main effect of group or time was significant without interaction, we performed an exploratory and limited pairwise post-hoc comparison, consistent with our a priori hypothesis. All statistical analyses using GraphPad Prism 7 (GraphPad Software, San Diego, CA, USA) were performed by investigators other than the experimenters to avoid bias and ensure blinding.

### Ethics approval and consent to participate

All experiments were approved by the Ethics Committee of Animal Experiments at the International University of Health and Welfare (Ohtawara, Japan; approval number: 19015, 22009). All animal experiments complied with the ARRIVE guidelines and were performed in accordance with the guidelines established by the Animal Research Committee of the International University of Health and Welfare and the United States National Institutes of Health Guide for the Care and Use of Laboratory Animals. Efforts were made to minimize the suffering of the animals and reduce the number of animals used in the experiments.

## Results

### Concentration-dependent effect of DSS on DAI scores, colon length, and anxiety-related behaviors in mice

The DAI scores for both stool consistency and rectal bleeding in DSS-treated mice (1.5%, 2%, or 2.5%) were significantly higher compared with those in the control group [Kruskal–Wallis test: p < 0.0001, Fig. 1b, p < 0.0001, Fig. 1c]. The colon length in DSS-treated mice (1.5%, 2%, or 2.5%) was significantly shorter compared to that in control group mice [one-way ANOVA: F (3, 52) = 300.6, p < 0.0001, Fig. 1d]. In the hole-board test, compared to the control group, the 2% DSS-treated group showed a significant decrease in the number of head-dips and the time spent in the central area (Fig. 1e,g), and the 2.5% DSS-treated group showed a significant decrease in performance across all items: the number or duration of head-dips and total time spent in the central area [one-way ANOVA: F (3, 52) = 5.034, p = 0.0039, Fig. 1e; F (3, 52) = 3.321, p = 0.0267, Fig. 1f; F (3, 52) = 14.56, p < 0.0001, Fig. 1g]. Furthermore, in the elevated plus-maze test, there were significant reductions in the number of open-arm entries as well as the percentage of distance traveled or time spent in the open arm in the 2% and 2.5% DSS treatment groups, compared to the control group [one-way ANOVA: F (3, 52) = 24.38, p < 0.0001, Fig. 1h; F (3, 52) = 21.71, p < 0.0001, Fig. 1i; F (3, 52) = 7.943, p = 0.0002, Fig. 1j].

Mice treated with 2.5% DSS were observed to move less, based on the distance covered, than controls (Supplementary Fig. S1). To exclude the possibility of decreased locomotor activity associated with exacerbated IBD-like symptoms, the concentration of 2% DSS was used in subsequent experiments in this study of anxiety associated with IBD.

### Time-dependent effects of DSS on DAI scores, colon length, and anxiety-related behaviors in mice

**Figure 2:**
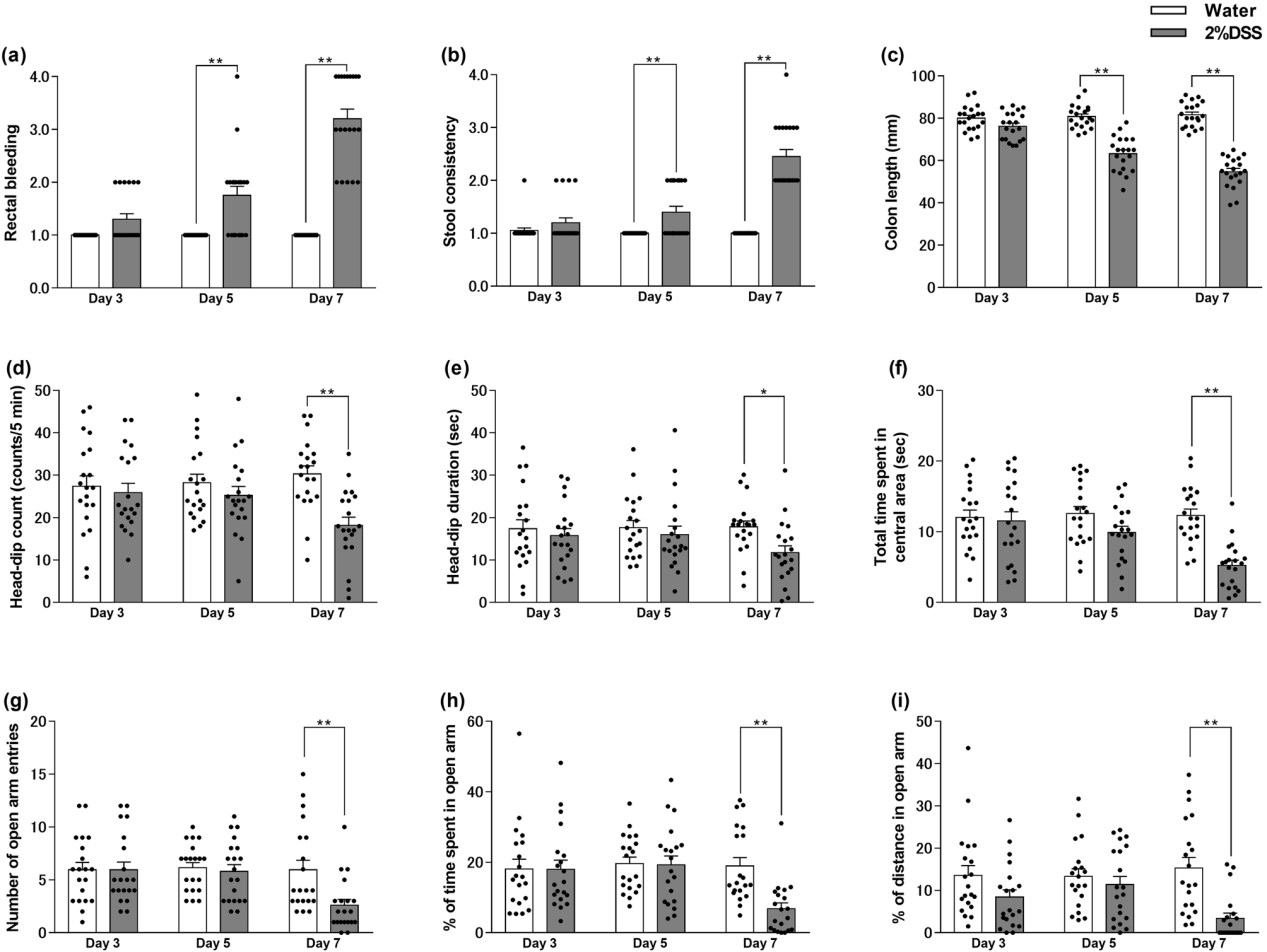
Time-course of DSS treatment for ulcerative colitis-like findings and anxiety-like behaviors. Time-course of rectal bleeding (**a**), stool consistency (**b**), colon length (**c**), head-dip counts (**d**), head-dip duration (**e**), total time spent in the central area (**f**), number of open-arm entries (**g**), percentage of distance traveled (**h**) or time spent (**i**) in the open arm in dextran sulfate sodium (DSS; 1.5%)-treated mice on days three, five, and seven. Bars represent means ± standard error of mean (SEM). *p < 0.05 and **p < 0.01 vs. water group (n = 20 per group).

As shown in Fig. 2, diarrhea, bloody stool, and shortened colon length were observed on days five and seven of 2% DSS treatment, but not on day three [two-way ANOVA: time: F (2, 114) = 38.55, p < 0.0001, group: F (1, 114) = 137.7, p < 0.0001, time × group: F (2, 114) = 38.55, p < 0.0001, Fig. 2a; time: F (2, 114) = 30.65, p < 0.0001, group: F (1, 114) = 95.6, p < 0.0001, time × group: F (2, 114) = 34.12, p < 0.0001, Fig. 2b; time: F (2, 114) = 23.14, p < 0.0001, group: F (1, 114) = 177.2, p < 0.0001, time × group: F (2, 114) = 30.68, p < 0.0001, Fig. 2c]. In contrast, reductions in the number or duration of head-dips, the time spent in the central area, the number of open-arm entries, and the percentage of distance traveled or time spent in the open arm were observed on day seven of 2% DSS treatment, but not on days three and five [two-way ANOVA: time: F (2, 114) = 0.914, p = 0.4038, group: F (1, 114) = 10.13, p = 0.0019, time × group: F (2, 114) = 3.74, p = 0.0267, Fig. 2d; time: F (2, 114) = 0.8254, p = 0.4407, group: F (1, 114) = 4.778, p = 0.0309, time × group: F (2, 114) = 1.083, p = 0.3420, Fig. 2e; time: F (2, 114) = 5.197, p = 0.0069, group: F (1, 114) = 17.91, p < 0.0001, time × group: F (2, 114) = 5.674, p = 0.0045, Fig. 2f; time: F (2, 114) = 3.989, p = 0.0212, group: F (1, 114) = 4.793, p = 0.0306, time × group: F (2, 114) = 3.561, p = 0.0316, Fig. 2g; time: F (2, 114) = 4.482, p = 0.0134, group: F (1, 114) = 4.952, p = 0.0280, time × group: F (2, 114) = 4.402, p = 0.0144, Fig. 2h; time: F (2, 114) = 1.196, p = 0.3062, group: F (1, 114) = 15.85, p = 0.0001, time × group: F (2, 114) = 3.353, p = 0.0384, Fig. 2i]. Based on these results, day seven after the commencement of 2% DSS treatment was found to be the best time point to investigate changes in the IBD model with anxiety.

### Effects of DZP or TDS on anxiety-related behaviors in 2% DSS-treated mice

**Figure 3:**
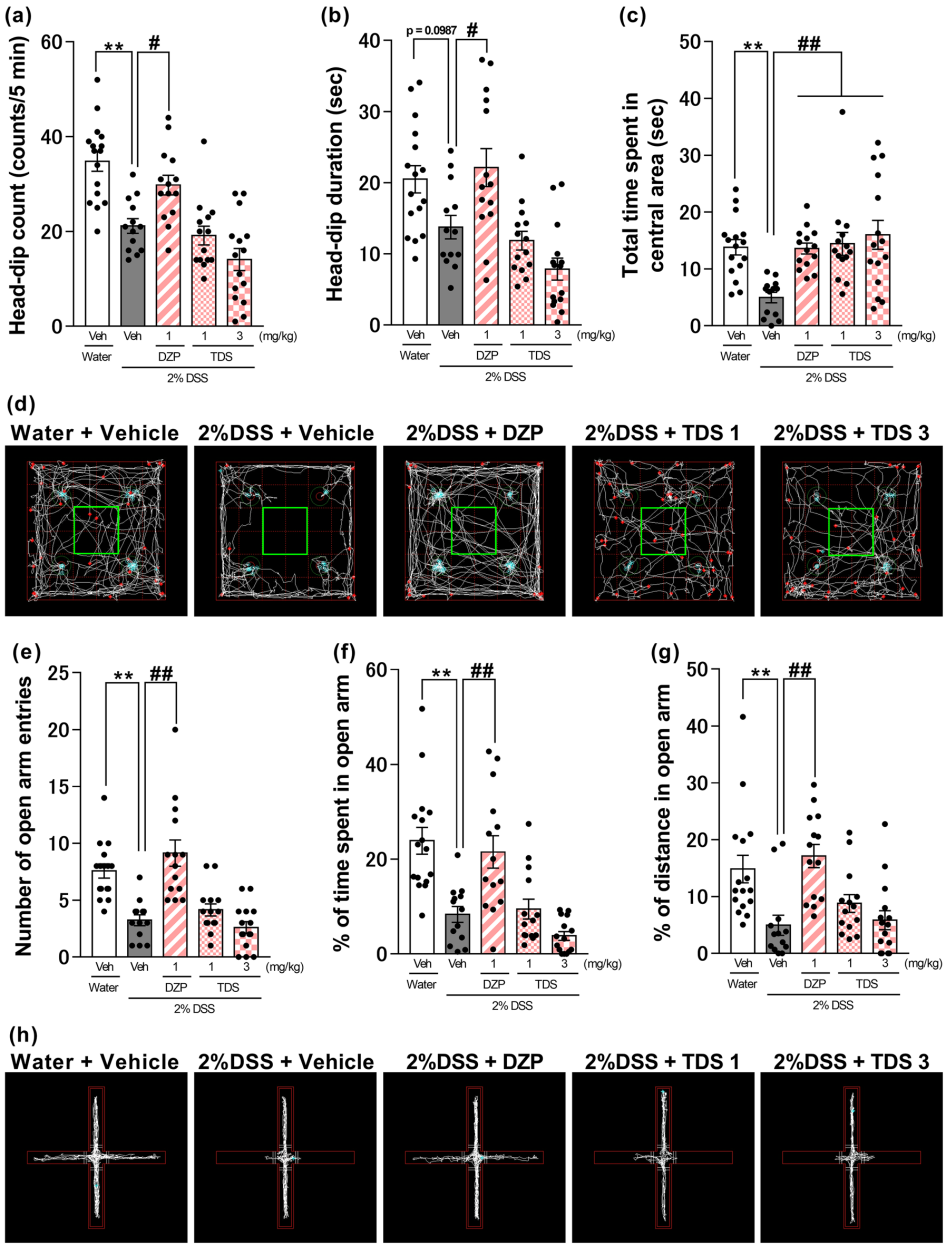
Effects of anxiolytics on DSS-induced anxiety-like behaviors. Effect of acute treatment with diazepam (DZP) or tandospirone (TDS) on head-dip counts (**a**), head-dip duration (**b**), total time spent in the central area (**c**), number of open-arm entries (**e**), percentage of distance traveled (**f**) or time spent (**g**) in the open arm in dextran sulfate sodium (DSS)-treated mice. (**d**) and (**h**) Representative activity traces in the hole-board test (**d**) and the elevated plus-maze test (**h**). The central area in the hole-board test is indicated by a green rectangle. Bars represent means ± standard error of mean (SEM). **p < 0.01 vs. water-treated water group, ^#^p < 0.05 and ^##^p < 0.01 vs. water-treated DSS group (n = 13–16 per group).

The effects of DZP and TDS—clinically used anxiolytic drugs—on anxiety-like behaviors in DSS-treated mice were examined. The results showed that acute administration of DZP (1 mg/kg) led to significant improvements in the outcomes assessed, effectively addressing reductions in the number or duration of head-dips, the time spent in the central area, the number of open-arm entries, and the percentage of distance traveled or time spent in the open arm in the 2% DSS-treated group in the hole-board and elevated plus-maze tests [one-way ANOVA: F (4, 67) = 17.52, p < 0.0001, Fig. 3a; F (4, 67) = 10.33, p < 0.0001, Fig. 3b; F (4, 67) = 5.942, p = 0.0004, Fig. 3c; F (4, 67) = 16.63, p < 0.0001, Fig. 3e; F (4, 67) = 14.12, p < 0.0001, Fig. 3f; F (4, 67) = 7.654, p < 0.0001, Fig. 3g]. In contrast, acute administration of TDS (1, 3 mg/kg) had a significant restorative effect only with regard to the decreased time spent in the central area, with no effect on other changes in the hole-board and elevated plus-maze tests (Fig. 3c,d).

### Changes in phosphorylation levels of GR in the brain of 2% DSS-treated mice

**Figure 4:**
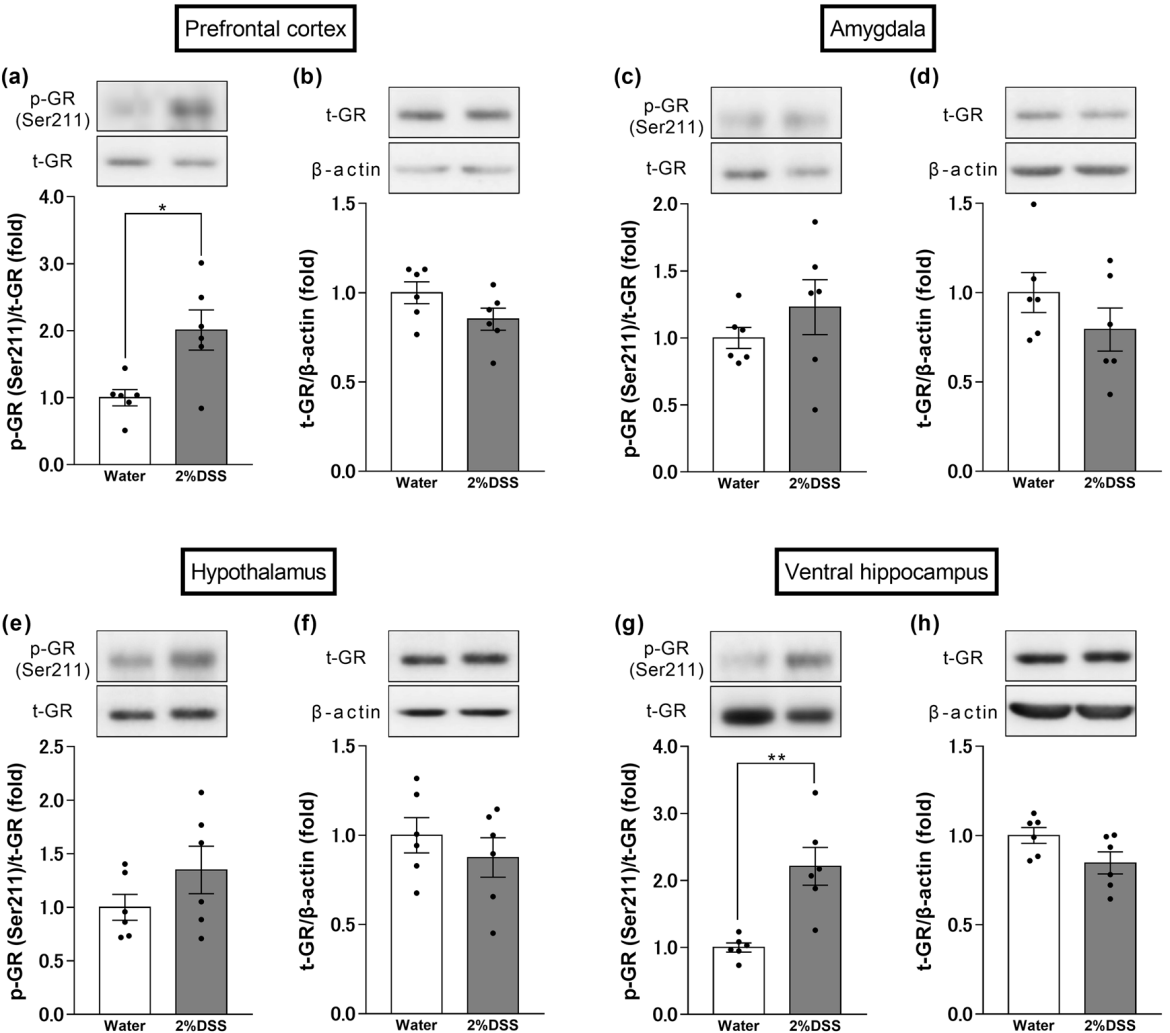
Changes in phosphorylation levels of glucocorticoid receptors in anxiety-related brain regions. Changes in phosphorylation and expression levels of glucocorticoid receptor (GR) in the prefrontal cortex (PFC) (**a**,**b**), amygdala (**c**,**d**), hypothalamus (**e**,**f**), and ventral hippocampus (**g**,**h**) of DSS-treated mice. Quantification of the normalized values of p-GR and t-GR with t-GR and β-actin, respectively. Original immunoblot images were shown in Supplementary Fig. S2. Bars represent means ± standard error of mean (SEM). *p < 0.05 and **p < 0.01 vs. water group (n = 6 per group).

We examined the changes in phosphorylation levels of GR in the brain regions that are closely related to the development of anxiety, such as the PFC, amygdala, hypothalamus, and ventral hippocampus. According to the results, 2% DSS-treated mice showed an increase in phosphorylation levels of GR in the PFC and ventral hippocampus, but not in the amygdala and hypothalamus, compared to the water group (Student’s t-test: t = 3.125, df = 10, p = 0.0108, Fig. 4a; t = 1.709, df = 10, p = 0.1182, Fig. 4b; t = 1.05, df = 10, p = 0.3182, Fig. 4c; t = 1.255, df = 10, p = 0.2381, Fig. 4d; t = 1.379, df = 10, p = 0.1980, Fig. 4e; t = 0.8407, df = 10, p = 0.4201, Fig. 4f; t = 4.181, df = 10, p = 0.0019, Fig. 4g; t = 2.02, df = 10, p = 0.0710, Fig. 4h).

### Effects of EF-2001 on DAI scores, colon length, and anxiety-related behaviors in mice

**Figure 5:**
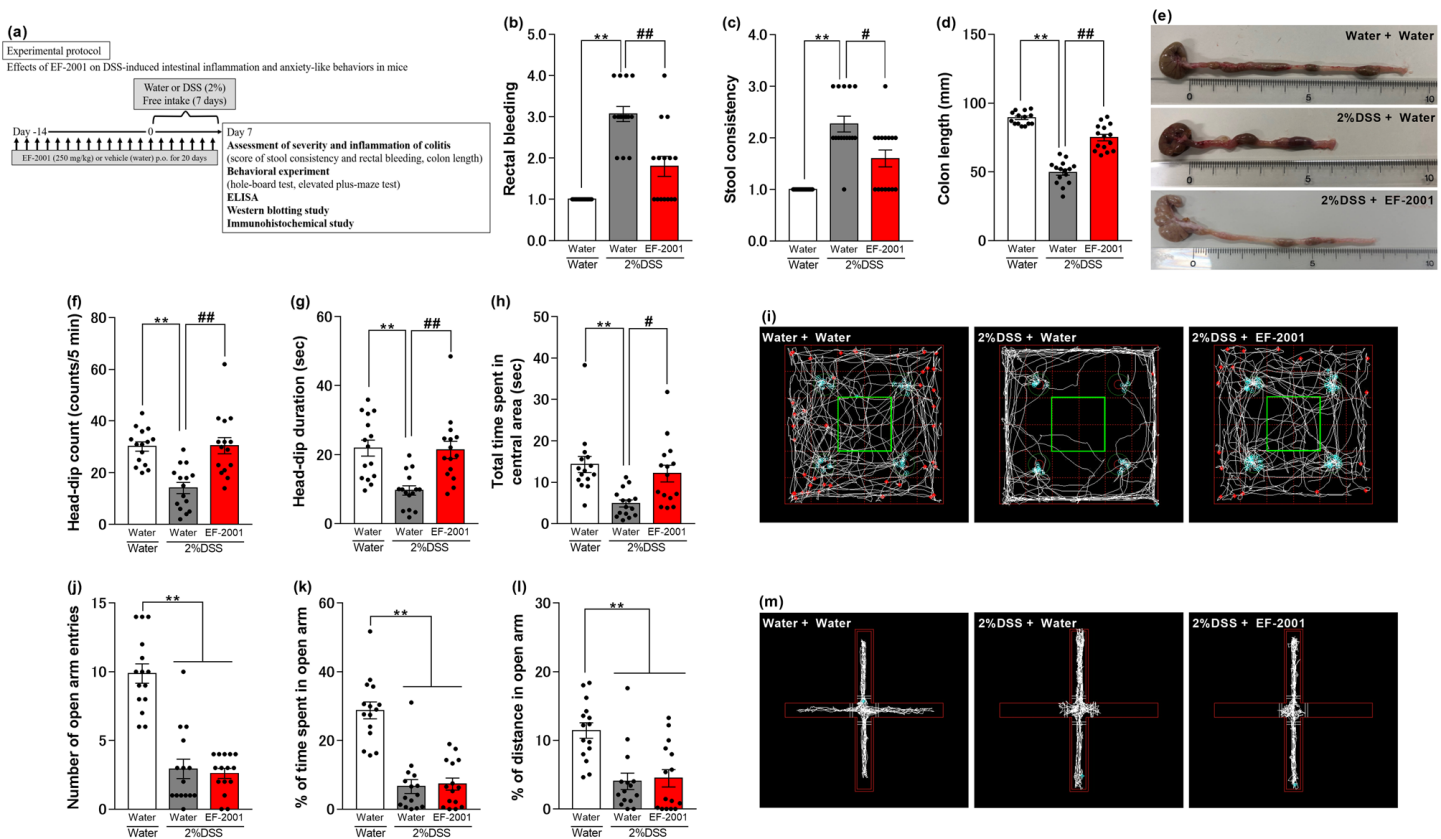
Effects of EF-2001 on DSS-induced ulcerative colitis-like findings and anxiety-like behaviors. (**a**) Experimental time course for assessment of inflammation, behavioral tests, western blotting, ELISA, and immunohistochemical of experimental protocols. Effects of chronic treatment with *Enterococcus faecalis* 2001 (EF-2001) on rectal bleeding (**b**), stool consistency (**c**), colon length (**d**), head-dip counts (**f**), head-dip duration (**g**), total time spent in the central area (**h**), the number of open-arm entries (**j**), percentage of distance traveled (**k**) or time spent (**l**) in the open arm in dextran sulfate sodium (DSS)-treated mice. (**e**) Representative colon images from each group are shown. (**i**) and (**m**) Representative activity traces in the hole-board test (**i**) and the elevated plus-maze test (**m**). The central area in the hole-board test is indicated by a green rectangle. Bars represent means ± standard error of mean (SEM). **p < 0.01 vs. water-treated water group, ^#^p < 0.05 and ^##^p < 0.01 vs. water-treated DSS group (n = 15 per group).

EF-2001 prevented DSS-induced diarrhea, bloody stool, and colon atrophy in mice [Kruskal–Wallis test: p < 0.0001, Fig. 5b, p < 0.0001, Fig. 5c. One-way ANOVA: F (2, 42) = 99.96, p < 0.0001, Fig. 5d]. Moreover, administration of EF-2001 in DSS-treated mice prevented reductions in the number or duration of head-dips and the time spent in the central area in the hole-board test [one-way ANOVA: F (2, 42) = 14.98, p < 0.0001, Fig. 5f; F (2, 42) = 10.82, p = 0.0002, Fig. 5g; F (2, 42) = 8.529, p = 0.0008, Fig. 5h]. In contrast, DSS-induced reductions in the number of open-arm entries and the percentage of distance traveled or time spent in the open arm in the elevated plus-maze test were not prevented by the administration of EF-2001 [one-way ANOVA: F (2, 42) = 44.88, p < 0.0001, Fig. 5j; F (2, 42) = 36.04, p < 0.0001, Fig. 5k; F (2, 42) = 37.68, p < 0.0001, Fig. 5l].

### Effects of EF-2001 on serum LPS and corticosterone concentration and p-GR in the brain

**Figure 6:**
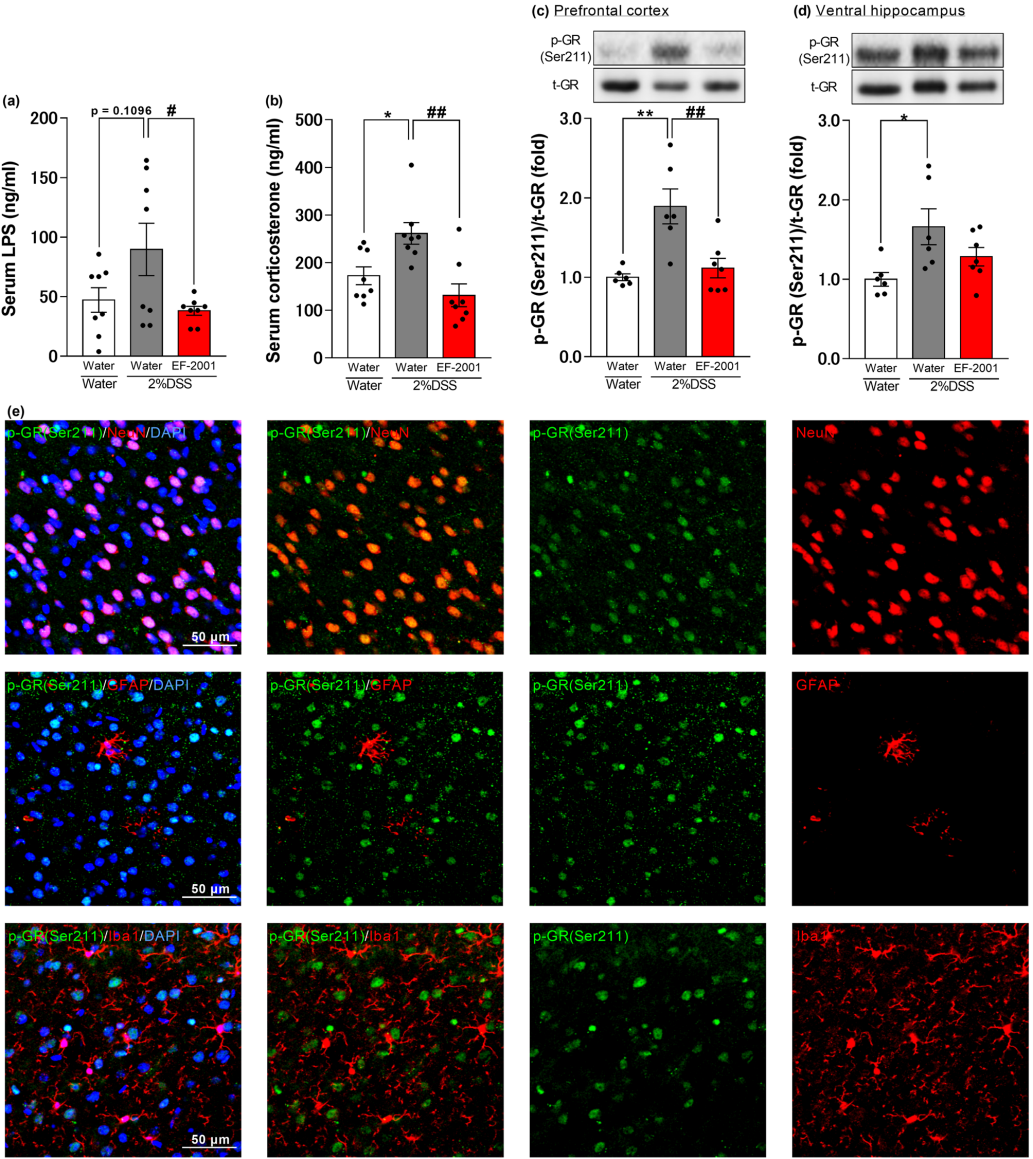
Effects of EF-2001 on serum LPS and corticosterone, and p-GR in the brain. Effects of chronic treatment with *Enterococcus faecalis* 2001 (EF-2001) on increased serum LPS (**a**) and corticosterone (**b**) concentration, and enhanced p-GR in the prefrontal cortex (PFC) (**c**) and ventral hippocampus (**d**) of DSS-treated mice. Quantification of the normalized values of p-GR with t-GR. Original immunoblot images were shown in Supplementary Fig. S3. (**e**) Microscopy images of p-GR (Ser211) (green), DAPI (blue), and NeuN, GFAP or Iba1 (red) immunostaining in the PFC of DSS-treated mice. Bars represent means ± standard error of mean (SEM). *p < 0.05 and **p < 0.01 vs. water-treated water group, ^#^p < 0.05 and ^##^p < 0.01 vs. water-treated DSS group (n = 6–8 per group).

Serum corticosterone levels in the 2% DSS-treated mice were significantly elevated compared to those in the water group (serum LPS concentration in the 2% DSS-treated group was slightly higher (p = 0.1096) compared to that in the water group), while these changes were prevented by EF-2001 administration [one-way ANOVA: F (2, 21) = 3.775, p = 0.0398, Fig. 6a; F (2, 21) = 9.151, p = 0.0014, Fig. 6b]. Moreover, administration of EF-2001 suppressed the increase of p-GR (Ser211) levels in the PFC of 2% DSS-treated mice, but not in the ventral hippocampus [one-way ANOVA: F (2, 16) = 10.78, p = 0.0011, Fig. 6c; F (2, 16) = 4.528, p = 0.0277, Fig. 6d]. Additionally, we observed that the p-GR (Ser211) labeling was colocalized in the neurons, but not in the astrocytes and microglia, of the PFC of DSS-treated mice (Fig. 6e).

### Effects of EF-2001 on NMDAR signaling and synaptic plasticity-related proteins

**Figure 7:**
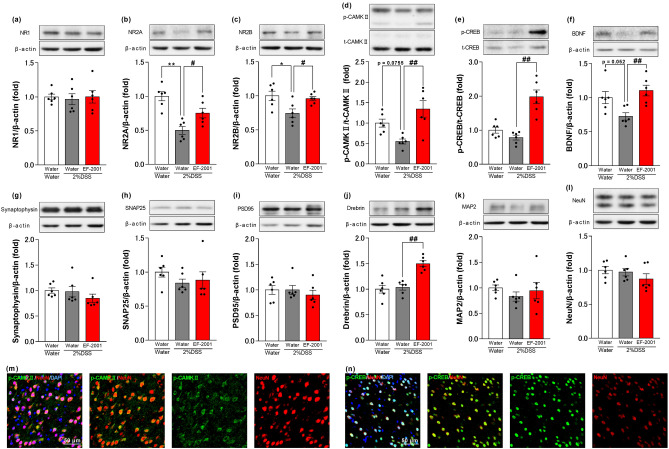
Effects of EF-2001 on NMDAR signaling and synaptic plasticity-related proteins in the PFC. Effects of chronic treatment with *Enterococcus faecalis* 2001 (EF-2001) on the NMDA receptor subunits (NRs), CAMKII/CREB/BDNF pathway, and synaptic plasticity-related proteins. a-l: Quantification of the normalized values of NR1 (**a**), NR2A (**b**), NR2B (**c**), BDNF (**f**), synaptophysin (**g**), SNAP25 (**h**), PSD95 (**i**), drebrin (**j**), MAP2 (**k**), and NeuN (**l**) levels with β-actin and p-CAMKII (**d**) and p-CREB (**e**) with t-CAMKII and t-CREB, respectively. Original immunoblot images were shown in Supplementary Fig. S4. (**m**) and (**n**) Microscopy images of NeuN (red), DAPI (blue), and p-CAMKII (**m**) or p-CREB (**n**) (green) immunostaining in the PFC of DSS mice treated with EF-2001. Bars represent means ± standard error of mean (SEM). *p < 0.05 and **p < 0.01 vs. water-treated water group, ^#^p < 0.05 and ^##^p < 0.01 vs. water-treated DSS group (n = 6 per group).

As shown in Fig. 7, the expression levels of NR2A and NR2B in the PFC of 2% DSS-treated mice were significantly lower compared to those in the water group (the expression levels of p-CAMKII and BDNF in the PFC of the 2% DSS-treated group decreased slightly (p-CAMKII: p = 0.0755; BDNF: p = 0.052) compared to those in the water group), whereas these changes were reversed by EF-2001 administration [one-way ANOVA: F (2, 15) = 15.01, p = 0.0003, Fig. 7b; F (2, 15) = 5.715, p = 0.0143, Fig. 7c; F (2, 15) = 8.886, p = 0.0028, Fig. 7d; F (2, 15) = 6.756, p = 0.0081, Fig. 7f]. Furthermore, EF-2001 treatment increased the expression levels of p-CREB and drebrin in the PFC of 2% DSS-treated mice [one-way ANOVA: F (2, 15) = 23.33, p < 0.0001, Fig. 7e; F (2, 15) = 19.03, p < 0.0001, Fig. 7j]. The expression levels of NR1, synaptophysin, SNAP25, PSD95 MAP2, and NeuN were not significantly different among the three groups [one-way ANOVA: F (2, 15) = 0.07509, p = 0.9280, Fig. 7a; F (2, 15) = 1.015, p = 0.3861, Fig. 7g; F (2, 15) = 0.8834, p = 0.4338, Fig. 7h; F (2, 15) = 0.4222, p = 0.6632, Fig. 7i; F (2, 15) = 0.6027, p = 0.5601, Fig. 7k; F (2, 15) = 1.181, p = 0.3339, Fig. 7l]. Additionally, we observed that the p-CAMKII and p-CREB labeling were colocalized in the neurons of the PFC (Fig. 7m,n).

## Discussion

A high prevalence of psychiatric disorders such as depression and anxiety has been reported among patients with IBD, but the mechanisms underlying the relationship between intestinal inflammation and anxiety symptoms remain unclear. In this study, we examined the effects of EF-2001 on IBD-like physiological changes and anxiety-like behaviors in DSS-treated mice. The results revealed that chronic administration of EF-2001 suppressed these changes. Furthermore, EF-2001 administration in DSS-treated mice suppressed the increase in serum LPS and corticosterone, the increase in GR phosphorylation, and the decrease in NR2A and NR2B expression in the PFC, and promoted the CAMKII/CREB/BDNF-Drebrin pathways in the PFC of DSS-treated mice. To the best of our knowledge, this is the first study to show that the anxiolytic effect of EF-2001 may be involved in the suppression of GR activity in the PFC via reduction of colon-derived LPS release and in synaptic plasticity via promotion of the CAMKII/CREB/BDNF-Drebrin pathways.

As mentioned above, a high rate of neuropsychiatric disorders has been reported to occur in patients with IBD^1–5^, but the underlying mechanisms remain unclear; other studies have reported that DSS-treated mice, commonly used in modeling UC, show UC findings, such as diarrhea and bloody stools, and exhibit anxiety-like behaviors^28,29^. In the present study, 2% DSS-treated mice exhibited UC-like symptoms such as diarrhea, hematochezia, and shortened colon length as well as anxiety-like behaviors in the hole-board and elevated plus-maze tests (Figs. 1, 2), without affecting locomotor activity (Supplementary Fig. S1). The effects of the clinically used anxiolytics DZP and TDS on anxiety-like behaviors in DSS-treated mice were also examined. The results showed that acute administration of DZP suppressed anxiety-like behaviors observed in the 2% DSS-treated group in both the hole-board and elevated plus-maze tests, while acute administration of TDS suppressed only the decrease in behavioral time in the central area in the hole-board test in the 2% DSS-treated group, but had no effect on other anxiety-like behaviors (Fig. 3). In clinical practice, TDS is less effective as a single dose than DZP and more effective when administered chronically; the results of this study appear to reflect this clinical fact^57^. Activation of GR in the PFC, hypothalamus, amygdala, and ventral hippocampus is known to induce anxiety-like behaviors via diverse mechanisms^12–15^. DSS-treated mice showed increased phosphorylation levels of GR in the PFC and ventral hippocampus (Fig. 4). From these results, we suggest that DSS-treated mice are a useful model of anxiety accompanying UC, as demonstrated the three validities (surface, predictive, and construct validities) which must be fulfilled when building a model animal^58,59^. Moreover, it has been suggested that increased phosphorylation of GR in the PFC and ventral hippocampus may be implicated in anxiety-like behaviors.

Previous studies demonstrated that EF-2001 administration prevented DSS-induced depression-like behavior and UC-like symptoms, with inflammation suppressed in the hippocampus and rectum^32^. In the present study, EF-2001 prevented DSS-induced UC-like symptoms and anxiety-like behaviors in the hole-board test, while it did not suppress anxiety-like behaviors in the elevated plus-maze test (Fig. 5). Moreover, administration of EF-2001 showed no changes in normal mice in both the hole-board and elevated plus-maze tests (Supplementary Fig. S5). As the elevated plus-maze test is thought to assess anxiety-like behavior under strong fear conditions compared to the hole-board test^60,61^, it may be that EF-2001 administration does not mitigate anxiety-like behavior associated with fear, but only that caused by mild stress.

In patients with UC, the intestinal microbiota is thought to be imbalanced, with a relative increase in gram-negative bacteria and a decrease in tight junctions between cells in the intestinal tract, leading to increased migration of inflammatory cytokines and LPS secreted by gram-negative bacteria into the blood^7,8^. In animal studies, intraperitoneal administration of LPS has been reported to increase blood corticosterone^9–11^. In the present study, DSS-treated mice were found to have increased serum LPS levels and a significant increase in serum corticosterone levels, and these changes were significantly prevented by EF-2001 administration (Fig. 6a,b). These results suggest that UC-like symptoms caused LPS to migrate from the intestinal tract into the blood and elevated the blood corticosterone levels. Moreover, EF-2001 administration prevented an increase in p-GR levels in the PFC of DSS-treated mice, but not in the ventral hippocampus (Fig. 6c,d). We found that phosphorylation of GR was localized to neurons in the PFC, but not to astrocytes and microglia (Fig. 6e). The ventral hippocampus region is known to be related to anxiety under conditions involving fearful stimuli, which can lead to high stress^62,63^. In the present study, EF-2001 showed no anxiolytic effect in the elevated plus-maze test, which assesses anxiety under fearful conditions. This result may be related to the fact that EF-2001 did not suppress the increase in phosphorylation levels of GR in the ventral hippocampus. From these findings, we suggest that the anxiolytic effect of EF-2001 may be associated with the decreased phosphorylation level of GR in the PFC neurons via the prevention of the increase in serum LPS and corticosterone levels.

Activation of GR by long-term stress exposure has been reported to cause abnormal synaptic plasticity^16^. Increased corticosterone causes decreased expression levels of NR2A, NR2B, BDNF, and synaptic plasticity-related proteins^17–19^, and these changes in the PFC are associated with the development of anxiety-like behavior^20–22^. Activated NMDAR phosphorylates CAMKII, which in turn phosphorylates CREB, resulting in BDNF and drebrin translation through CREB activation^23–25^. Activation of BDNF signaling by the tropomyosin receptor kinase B agonist 7,8-dihydroxyflavone produces anxiolytic effects^26^. It has been reported that increased expression of drebrin in the PFC plays an important role in anxiolytic effects^27^. BDNF and Drebrin expression in the PFC has been suggested to correlate with anxiety-like behaviors in rodents^27,64,65^. In the present study, the expression levels of p-CAMKII and BDNF in the PFC of DSS-treated mice showed a decreasing tendency, and the expression levels of NR2A and NR2B significantly decreased, while these changes were reversed by EF-2001 treatment (Fig. 7b–d,f). Furthermore, EF-2001 treatment increased the expression levels of p-CREB and drebrin in the PFC of DSS-treated mice (Fig. 7e,j). In contrast, the expression levels of NR1, synaptophysin, SNAP25, PSD95, MAP2, and NeuN in the PFC were not significantly altered. We found that p-CAMKII and p-CREB were localized to neurons in the PFC (Fig. 7m,n). From these findings, we suggest that the anxiolytic effects of EF-2001 may be associated with activation of the CAMKII/CREB/BDNF-Drebrin pathways in the PFC neurons via normalized expression levels of NR2A and NR2B.

Despite these novel results, this study had some limitations that must be noted. In the present study, we focused on the relationship between anxiety behavior and activation of GR in the brain. However, we consider that studies focusing on these relationships alone are insufficient to elucidate the pathogenesis of anxiety accompanying UC. The reason is that there have been many studies focusing on neural activity in the brain and anxiety behavior^66–69^, which we did not examine in this study. Moreover, it is unclear as to how elevated serum LPS contributes to elevated corticosterone and how EF-2001 acts on changes in serum LPS. Furthermore, it has been reported that changes in the intestinal microbiota affect the brain via the vagus nerve, resulting in anxiety-like behavior^70^. To determine whether the microbiota-gut-brain axis via the vagus nerve contributes to the anxiolytic effects of EF-2001, it is necessary to examine changes in the gut microbiota and the effects of vagotomy. These issues will be clarified in future studies.

## Conclusion

**Figure 8:**
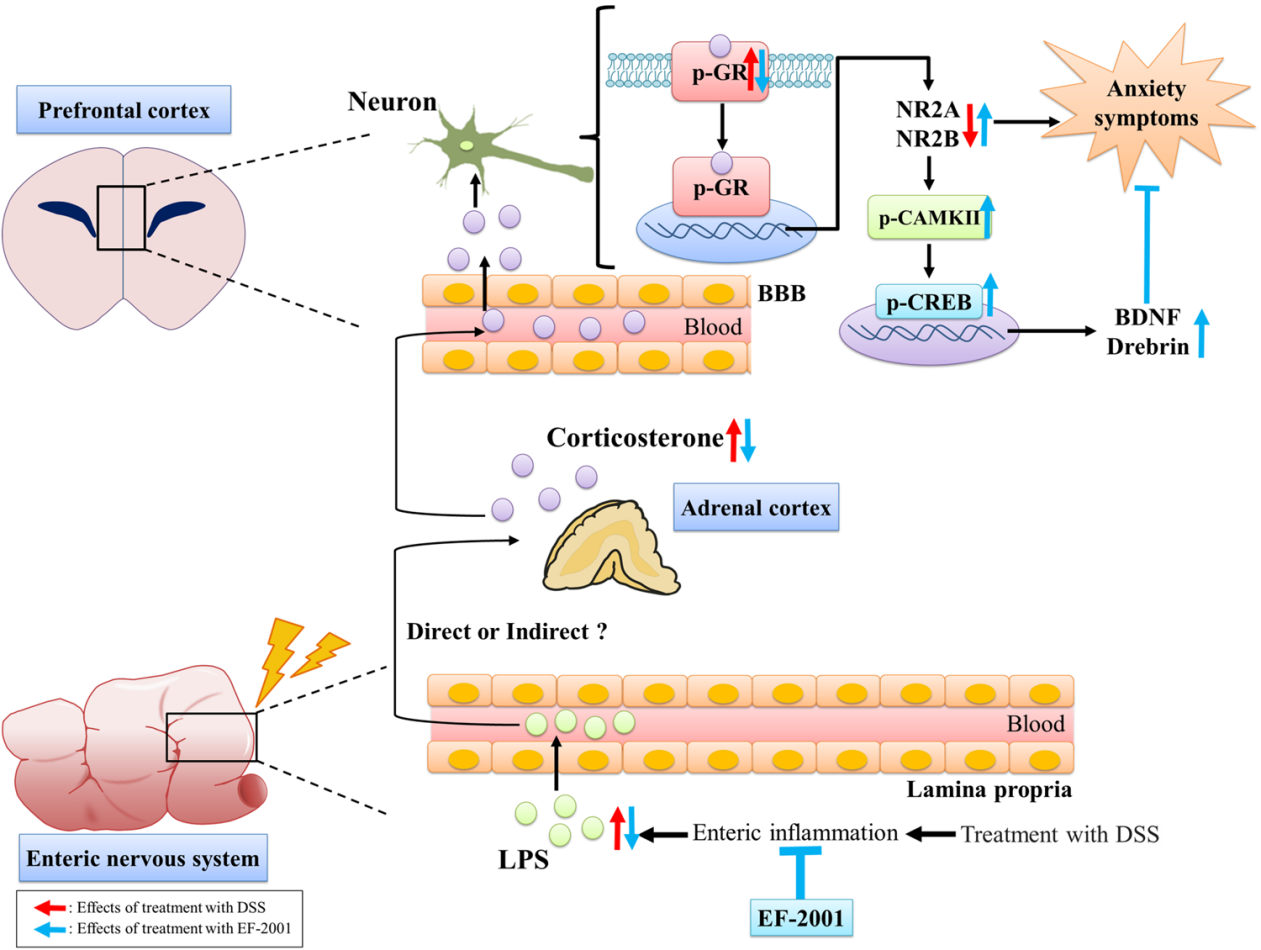
Mechanistic hypothesis for the anti-anxiety effects of EF-2001.

As summarized in Fig. 8, the results from our DSS-induced UC model with mice show that elevated serum LPS and corticosterone levels activate GR in the PFC, resulting in anxiety-like behaviors. EF-2001 inhibits these changes by preventing enteritis symptoms, and produces anxiolytic effects via the activation of synaptic plasticity in the PFC. These results suggest a close relationship between IBD and anxiety, and provide important evidence for a mechanism by which the attenuation of intestinal inflammatory symptoms in UC may reduce the risk of psychiatric disorders.

### Supplementary Information

Supplementary Information.

## Data Availability

The datasets used and/or analyzed in the current study are available from the corresponding author on reasonable request.

## Abbreviations

ANOVA: Analysis of variance
BDNF: Brain-derived neurotrophic factor
BRM: Biological response modifier
CAMKII: Calcium/calmodulin-dependent protein kinase II
CREB: CAMP response element binding protein
DAI: Disease activity index
DZP: Diazepam
DSS: Dextran sulfate sodium
EF-2001: Enterococcus Faecalis 2001
ELISA: Enzyme-linked immunosorbent assay
GFAP: Glial fibrillary acidic protein
GR: Glucocorticoid receptor
Iba1: Ionized calcium-binding adapter molecule 1
IBD: Inflammatory bowel disease
IgG: Immunoglobulin G
i.p.: Intraperitoneally
LPS: Lipopolysaccharides
MAP2: Microtubule-associated protein 2
NeuN: Neuronal nuclei
NMDAR: *N*-Methyl-d-aspartate receptor
NR: *N*-Methyl-d-aspartate receptor subunit
PBS: Phosphate-buffered saline
PBSBT: PBS containing 3% bovine serum albumin and 0.3% Triton X-100
PBSGT: PBS containing 1% normal goat serum and 0.3% Triton X-100
p.o.: Per os
PFC: Prefrontal cortex
PSD95: Postsynaptic density protein 95
SEM: Standard error of the mean
SNAP25: Synaptosomal-associated protein 25
TBST: Tris-buffered saline supplemented with 0.01% Tween-20
TDS: Tandospirone
UC: Ulcerative colitis
