# Supplementary material for: Anxiolytic effects of Enterococcus faecalis 2001 on a mouse model of colitis
Source: Sci Rep. 2024 May 21;14:11519. doi: 10.1038/s41598-024-62309-3 (PMC11106339; doi:10.1038/s41598-024-62309-3)
Supplement: Supplementary file 1 — Supplementary Information. [file 41598_2024_62309_MOESM1_ESM.docx]

**Supplemental information**

| **Score** | **Stool consistency** | **Rectal bleeding** |
| --- | --- | --- |
| **1** | **Normal** | **Absent** |
| **2** | **Loose stool**  (tangible stool with high moisture content) | **Visible blood stool** |
| **3** | **Diarrhea stool**  (collapsed stool with high moisture content) | **Blood is attached**  **to the anus** |
| **4** | **Watery diarrhea**  (almost intangible liquid stool) | **Always bleeding**  **from the anus** |

**Supplemental Table S1. Score of stool consistency and rectal bleeding.**


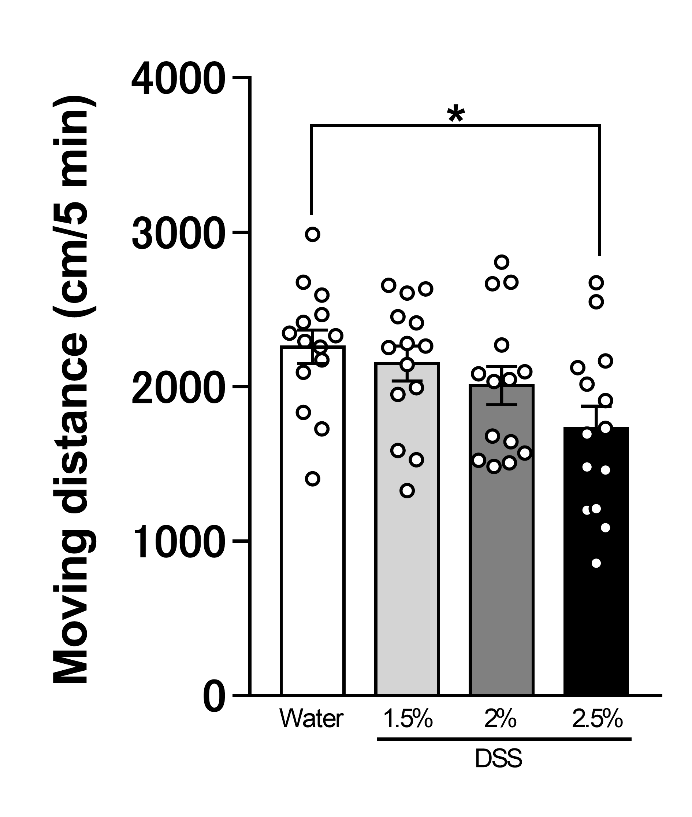


**Fig. S1. Changes in moving distance in dextran sulfate sodium (DSS)-treated mice on day seven.**

One-way ANOVA: F (3, 52) = 3.448, p = 0.0231, Fig. S1. Bars represent means ± standard error of mean (SEM). *p < 0.05 vs. water group (n = 14 per group).

**
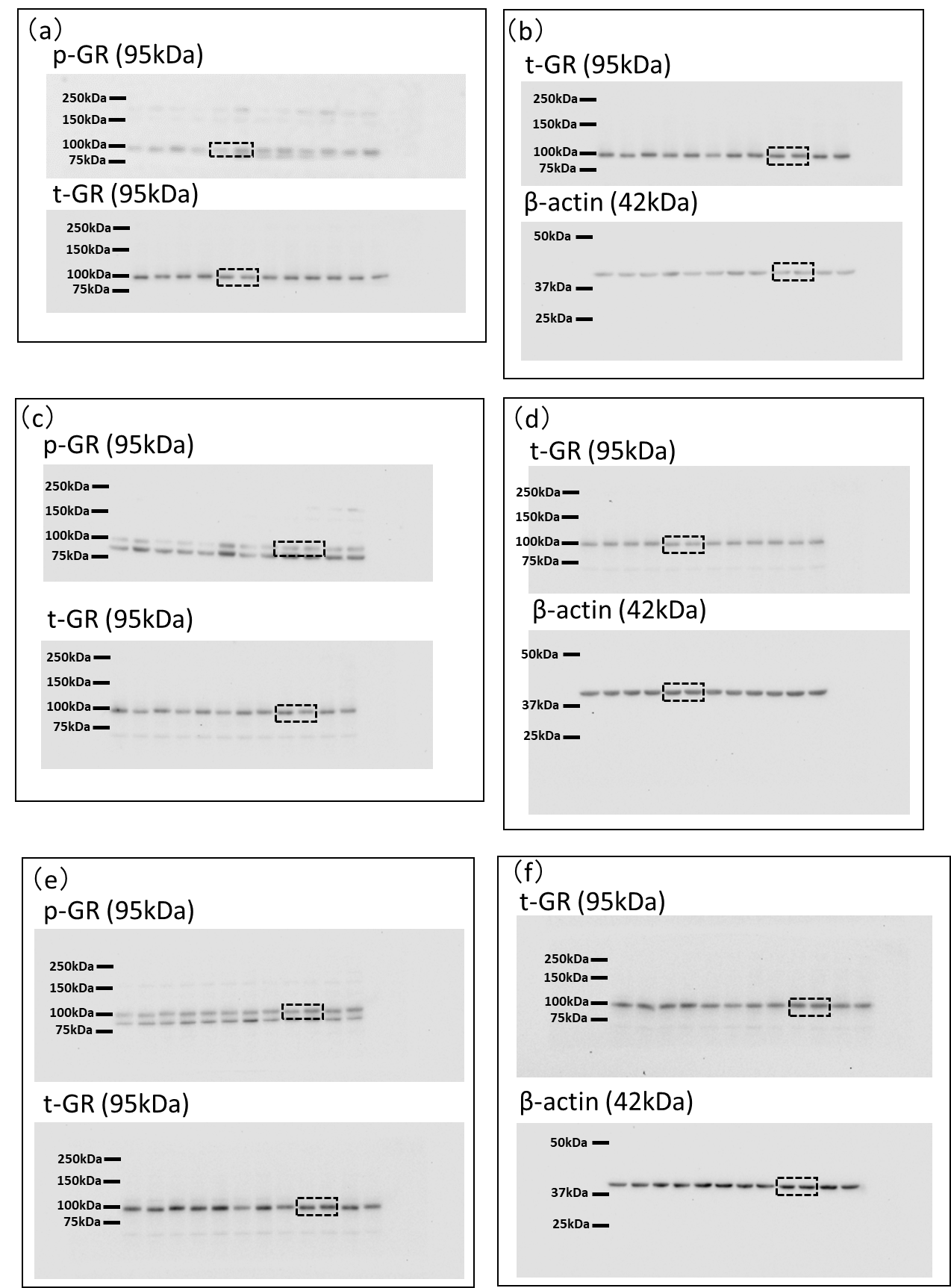
**

**
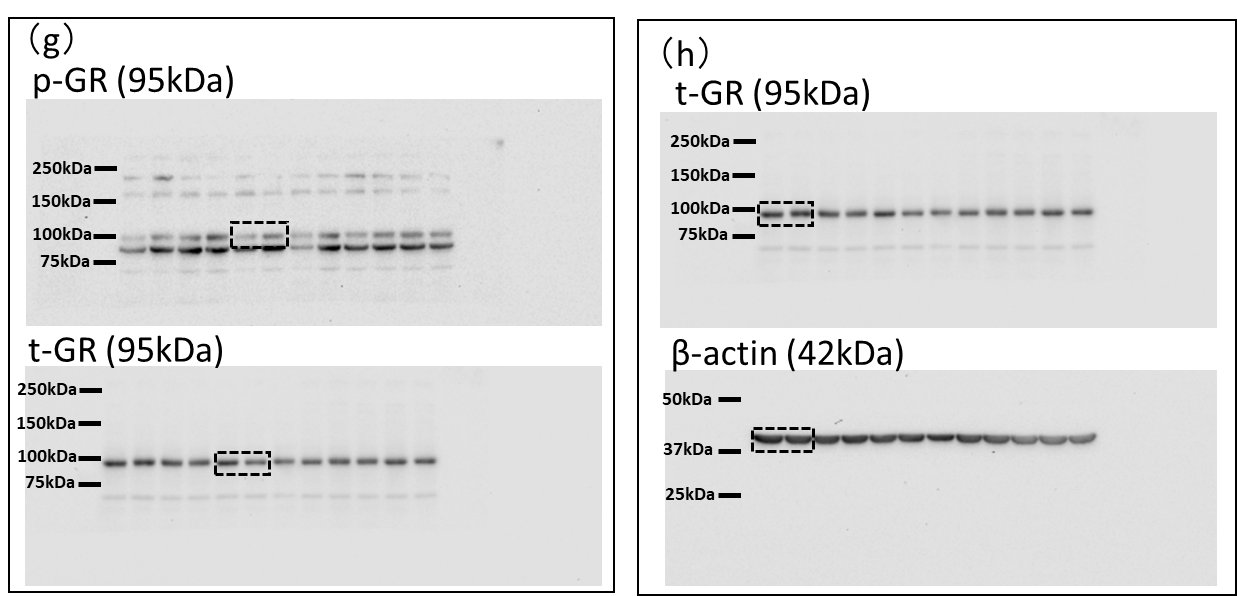
**

**Fig. S2. Original membrane and immunoblot images supplemental to Fig. 4a-4h.** Membranes were cut between 50kDa and 75kDa, and probed with antibodies.

**
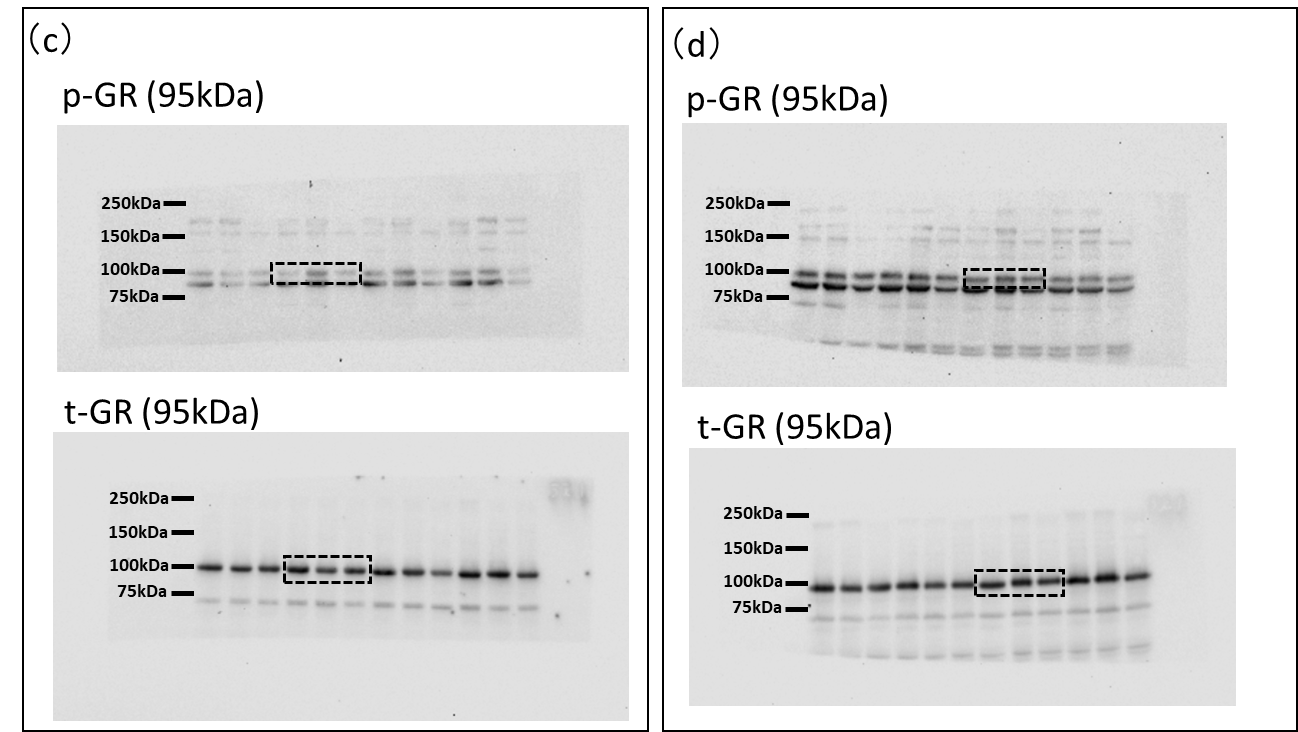
**

**Fig. S3. Original membrane and immunoblot images supplemental to Fig. 6c and 6d.** Membranes were cut between 50kDa and 75kDa, and probed with antibodies.

**
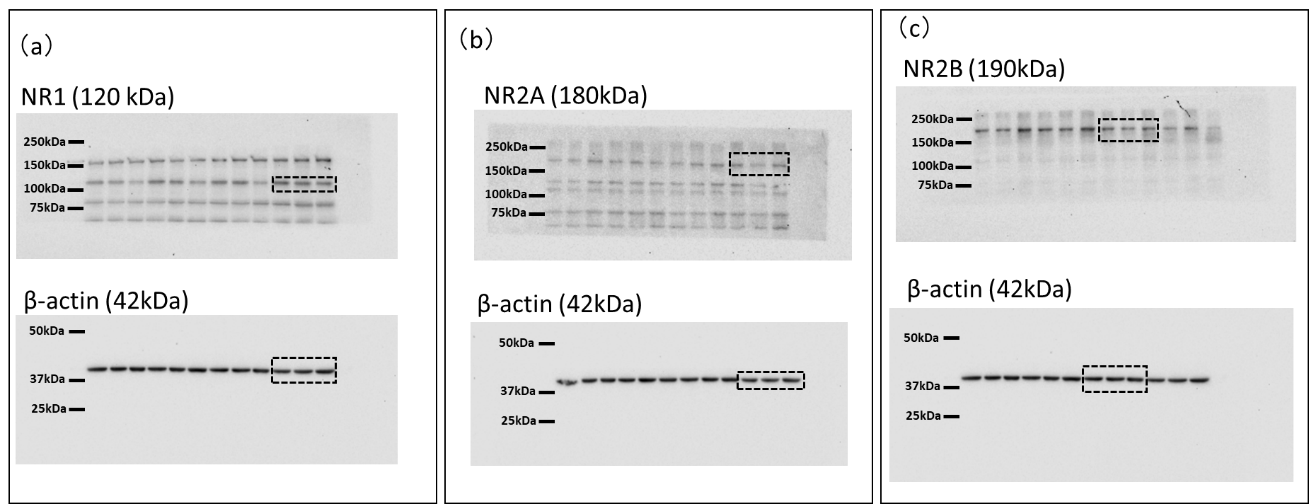

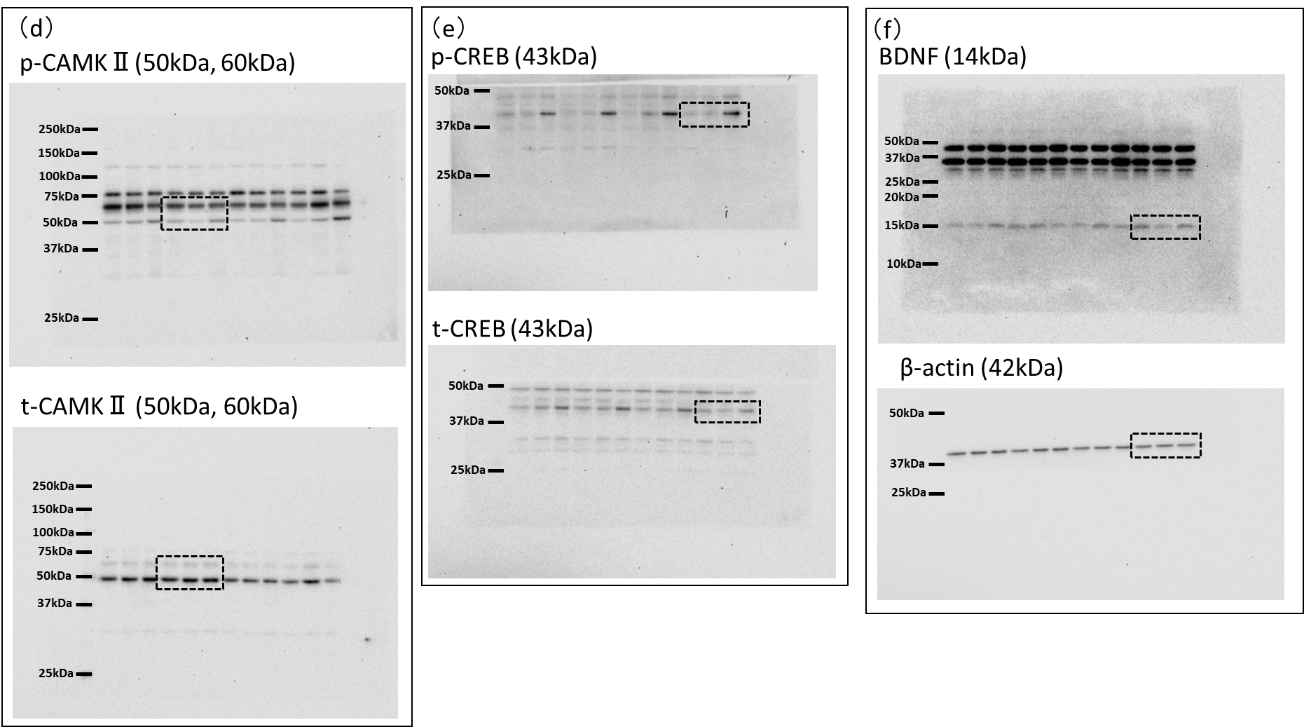

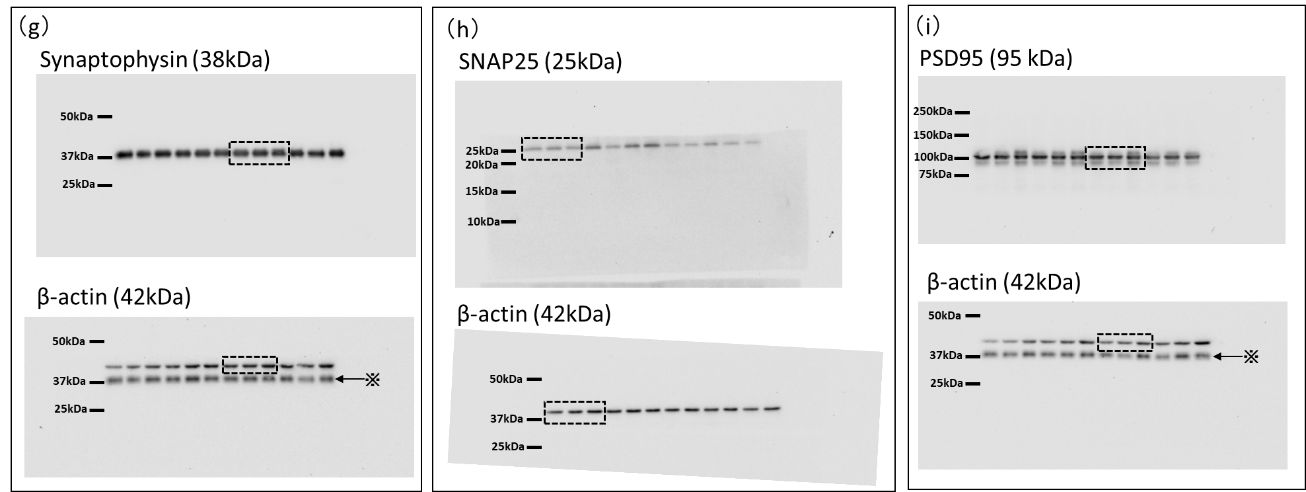
**

**
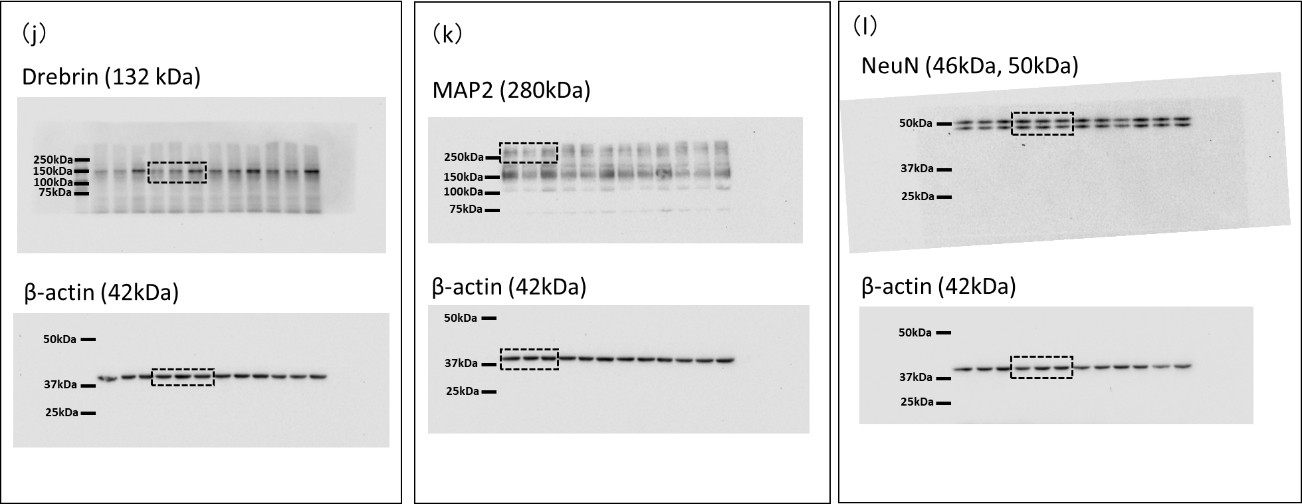
**

**Fig. S4. Original membrane and immunoblot images supplemental to Fig. 7a-7l.** Membranes were cut between 50kDa and 75kDa, and probed with antibodies except for p-CAMKⅡ, t-CAMKⅡ, and BDNF. ※: This band is synaptophysin that could not be stripped by ReBlot Plus Strong solution (Millipore, #2504).


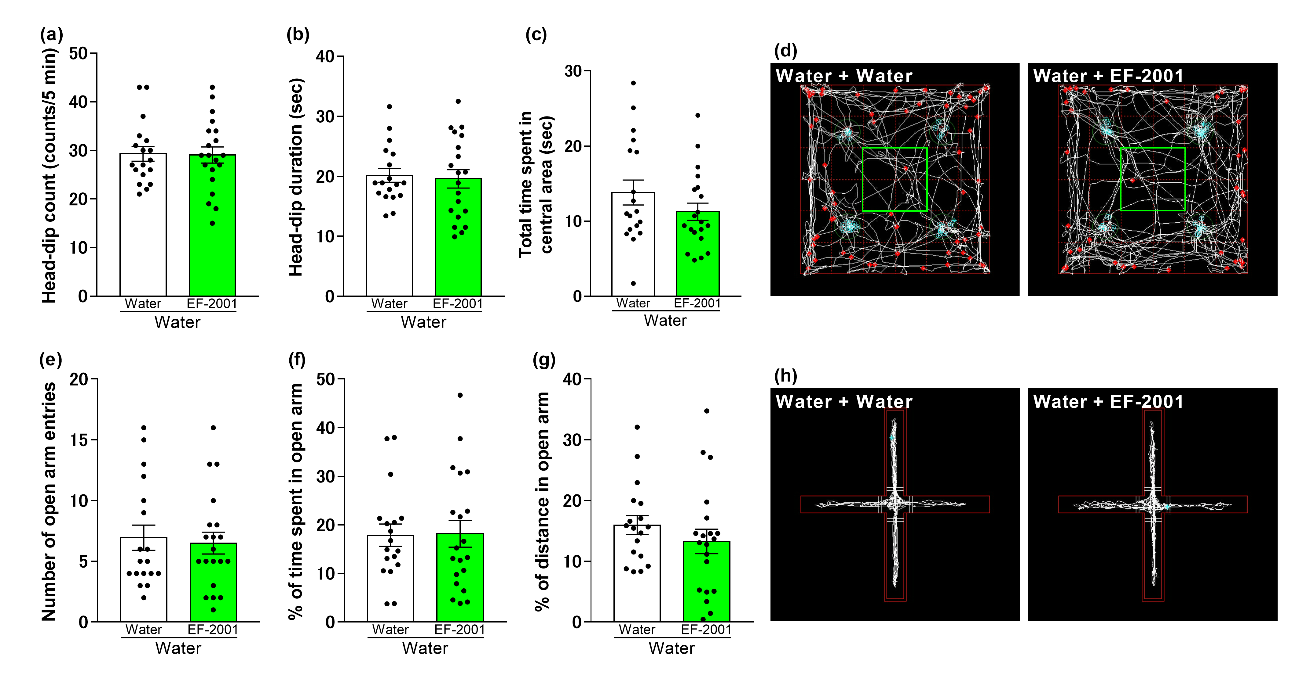


**Fig. S5.** **Effects of EF-2001 on anxiety-like behaviors in water-treated mice.**

Effects of chronic treatment with *Enterococcus faecalis* 2001 (EF-2001) on head-dip counts (a), head-dip duration (b), total time spent in the central area (c), number of open-arm entries (e), percentage of distance traveled (f) or time spent (g) in the open arm in water-treated mice. d and h: Representative activity traces in the hole-board test (d) and the elevated plus-maze test (h). The central area in the hole-board test is indicated by a green rectangle. Student’s t-test: t = 0.09997, df = 36, p = 0.9209, Fig. S5a; t = 0.2936, df = 36, p = 0.7707, Fig. S5b; t = 1.289, df = 36, p = 0.2056, Fig. S5c; t = 0.325, df = 36, p = 0.7471, Fig. S5e; t = 0.08244, df = 36, p = 0.9348, Fig. S5f; t = 1.036, df = 36, p = 0.3073, Fig.S5g. Bars represent means ± standard error of mean (SEM).
